# An analysis pipeline for understanding 6-thioguanine effects on a mouse tumour genome

**DOI:** 10.1007/s00262-023-03610-4

**Published:** 2024-01-27

**Authors:** Patricio Yankilevich, Loulieta Nazerai, Shona Caroline Willis, Kjeld Schmiegelow, Daniela De Zio, Morten Nielsen

**Affiliations:** 1 Bioinformatics Core Facility, Instituto de Investigación en Biomedicina de Buenos Aires (IBioBA) - CONICET - Partner Institute of the Max Planck Society, Buenos Aires, Argentina; 2 Melanoma Research Team, Danish Cancer Institute, Copenhagen, Denmark; 3 Department of Pediatrics and Adolescent Medicine, Copenhagen University Hospital - Rigshospitalet, Copenhagen, Denmark; 4 Department of Cancer and Inflammation Research, Institute of Molecular Medicine, University of Southern Denmark, Odense, Denmark; 5 Department of Health Technology, Section for Bioinformatics, Technical University of Denmark, Lyngby, Denmark

**Keywords:** Mouse genome analysis pipeline, Mouse tumour models, 6-Thioguanine (6TG) treatment, Immune-checkpoint inhibitors (ICI), Neoantigen analysis, Tumour mutational burden

## Abstract

Mouse tumour models are extensively used as a pre-clinical research tool in the field of oncology, playing an important role in anticancer drugs discovery. Accordingly, in cancer genomics research, the demand for next-generation sequencing (NGS) is increasing, and consequently, the need for data analysis pipelines is likewise growing. Most NGS data analysis solutions to date do not support mouse data or require highly specific configuration for their use. Here, we present a genome analysis pipeline for mouse tumour NGS data including the whole-genome sequence (WGS) data analysis flow for somatic variant discovery, and the RNA-seq data flow for differential expression, functional analysis and neoantigen prediction. The pipeline is based on standards and best practices and integrates mouse genome references and annotations. In a recent study, the pipeline was applied to demonstrate the efficacy of low dose 6-thioguanine (6TG) treatment on low-mutation melanoma in a pre-clinical mouse model. Here, we further this study and describe in detail the pipeline and the results obtained in terms of tumour mutational burden (TMB) and number of predicted neoantigens, and correlate these with 6TG effects on tumour volume. Our pipeline was expanded to include a neoantigen analysis, resulting in neopeptide prediction and MHC class I antigen presentation evaluation. We observed that the number of predicted neoepitopes were more accurate indicators of tumour immune control than TMB. In conclusion, this study demonstrates the usability of the proposed pipeline, and suggests it could be an essential robust genome analysis platform for future mouse genomic analysis.

**Supplementary Information:**

The online version contains supplementary material available at 10.1007/s00262-023-03610-4.

## Introduction

The use of mouse tumour models constitutes the most widely used pre-clinical research tool in oncology [1], having an important role in the discovery and development of anticancer drugs [2]. A critical novel approach from cancer treatment is immunotherapy, where the host immune system is boost to destroy cancer cells. Understanding how the immune system interacts with tumours is therefore crucial for developing personalised immunotherapies and cancer treatments. Cancer genomics research has been revolutionised by the advances in next-generation sequencing (NGS). With costs constantly dropping, the demand for sequencing of mouse cancers is increasing, as well as the need for robust analysis pipelines [3]. The development of most analytical tools and bioinformatics pipelines to analyse sequencing data, to date, have focussed on humans and hence do not account for species-specific differences in genome structures and experimental setups; and have so far not been systematically validated in the mouse context [3]. The genome analysis toolkit (GATK) is the gold standard in germline variant discovery. It was originally developed for human genetics, and only recently its scope has been expanding to include other organisms and somatic variant calling [4]. The GATK team is actively working on expanding access to other species, but the development and validation of new, robust, and reliable tools is not an easy process. The functional annotator tool in GATK, Funcotator, for instance, does not currently support non-human genomes. Although GATK was originally designed for human genome research, its best practices can be adapted to the analysis of non-human organisms. A critical challenge in developing a robust tumour genome analysis pipeline is to choose the appropriate analysis methods for somatic variant discovery and biomarker identification, but also the correct file formats, genome references and annotations. In this study, we propose a genome analysis pipeline designed specifically for mouse tumours. The pipeline encompasses three main components: a data analysis workflow for somatic variant discovery using whole-genome sequencing (WGS) data, a workflow for differential expression analysis using RNA-sequencing (RNA-seq) data, and a workflow for neoepitope prediction through neoantigen analysis. Neoepitopes are the MHC (major histocompatibility complex) presented targets for immune responses against cancer. The pipeline is based on standards and best practices, and is configured to integrate mouse references and annotations. All the file formats and genome references used, and all tools, methods, algorithms and packages included in our pipeline are current standards on NGS data analysis, and were quantitatively evaluated in regard to accuracy, precision, and reliability [5–8].

The thiopurine drugs are purine antimetabolites widely used in the treatment of haematological cancers, autoimmune disorders, and organ transplant recipients. The thiopurine thioguanine, also known as 6-thioguanine (6TG), is used to treat acute myeloid leukaemia, acute lymphocytic leukaemia, and chronic myeloid leukaemia [9]. Thiopurines are converted into thioguanine nucleotides that are incorporated into DNA in competition with normal guanine inducing mutations through single nucleotide mismatching [10]. We recently applied the pipeline to show that the treatment with low dosage of 6TG of low-mutation melanoma in a pre-clinical mouse model is highly effective in reactivating T cells to attack cancer and mildly increases the tumour mutational burden (TMB) [11]. Moreover, the combination of 6TG with the immune-checkpoint inhibitors (ICI), which block the interaction between the inhibitory receptors on T cells and their ligands, enhances the response to ICI therapy [11]. Here, we describe the pipeline applied in the study and further the results by analysing the potential for MHC class I antigen presentation of the identified tumour mutational space, and investigate of how this space correlates to tumour control.

## Materials and methods

All laboratory in vitro and in vivo experiments and assays performed in the context of this project are described in Nazerai et al*.* [11]. The materials and methods described include mice, mice monitoring-endpoints, cell preparation and proliferation, DNA content analysis, DNA-TG levels’ mass spectrometry, syngeneic melanoma model, histological analysis, in vivo T cell depletion, flow cytometry, and antibodies among other techniques. The following sections describe materials and methods for tumour sequencing and genome analysis and interpretation.

All pipelines were implemented on Computerome, the Danish National Computer for Life Sciences (https://www.computerome.dk/). This supercomputer has a High-Performance Computing (HPC) solution that provides a secure and powerful computer cluster where data can be analysed and stored.

### DNA extraction, whole-genome sequencing and RNA-sequencing

Tumour fractions were preserved in RNAlater Stabilization Solution (Thermo Fisher Scientific), according to the instructions. DNA extracted from Control-Yumm and 6TG-Yumm tumours as well as control YUMM cell lines using Qiagen’s DNeasy Blood and Tissue Kit were used for Whole-Genome Sequencing. After sample quality control and library preparation, sequencing was performed using the Illumina Novaseq 6000 platform.

### Genome analysis pipeline—WGS data flow

FastQC v0.11.9 was used to perform quality control checks on fastq files containing raw sequence data from tumour samples. The fastq files were aligned to the C57BL/6 J GRCm38 (mm10) mouse reference genome using the BWA-MEM algorithm from the Burrows–Wheeler Aligner tool v.0.7.17 [12]. Picard-tools v.2.26.106 and GATK v.4.2.5.0 [13] were used for BAM pre-processing. Somatic single nucleotide variants and indels were identified using the GATK’s Mutect2 [14] configured to run in somatic (tumour control) mode. The BAM file obtained from the alignment of non-treated Yumm cells WGS data was used as control. The identified somatic variants were annotated with ANNOVAR v.2019oct24 [15], using the mouse mm10 reference genes from UCSC as reference. Finally, the R package Maftools v.2.8.05 [16] was used to create a Mutation Annotation Format (MAF) file per sample and calculate the TMB, along with other statistics and graph plotting. The association between TMB and tumour volume was calculated by using the Pearson correlation coefficient, and *p* value obtained using exact permutation testing.

### Genome analysis pipeline—RNA-seq data flow

The RNA-seq fastq files were mapped using the STAR aligner v2.7.9a. [17]. The mouse genome build GRCm38.68 and the UCSC mm10 RefGene annotations were used as references. Samtools v1.14 [18] was used to index BAM files. Reads per gene were quantified using the STAR quantMode. Further filtering, normalisation and identification of differentially expressed genes were done with the R package DESeq2 v.1.32.0 [19]. Functional analysis was performed with GSEA v4.2.3 [20] and the gene set collections in the mouse Molecular Signature database (MSigDB) v2022.1.Mm, which includes 15,918 gene sets divided into 6 major collections, and several sub-collections. Gene set enrichments were tested running GSEA configured with different permutation type parameters (phenotypes and gene sets). Older gene sets from the database for pathway analysis in mouse Gene Set Knowledgebase (GSKB) from July 2013 (http://ge-lab.org/#/data) were also tested.

### Genome analysis pipeline—Neoantigen analysis flow

Tumour somatic mutation profiles (VCF file) and tumour gene expression data from every tumour sample were processed with MuPeXI v1.1.3 [21, 22] to obtain all unique mutated peptides (neopeptides) and, by calling NetMHCpan v4.0 [23], identify which are the neopeptides most likely to serve as neoepitopes. MuPeXI was configured with GRCm38 (mm10) mouse reference genome, and NetMHCpan MHC mouse alleles were configured with H-2-Kb and H-2-Db. The list of predicted neoepitopes was obtained by filtering the potential neopeptides with best binding affinity score (MHCrank < 5) and existing gene expression (expression level > 0). The association between the number of predicted neoepitopes and tumour volume was calculated by using the Pearson correlation coefficient, and *p* value obtained using exact permutation testing.

## Results

### Design and development of the genome analysis pipeline

**Fig. 1:**
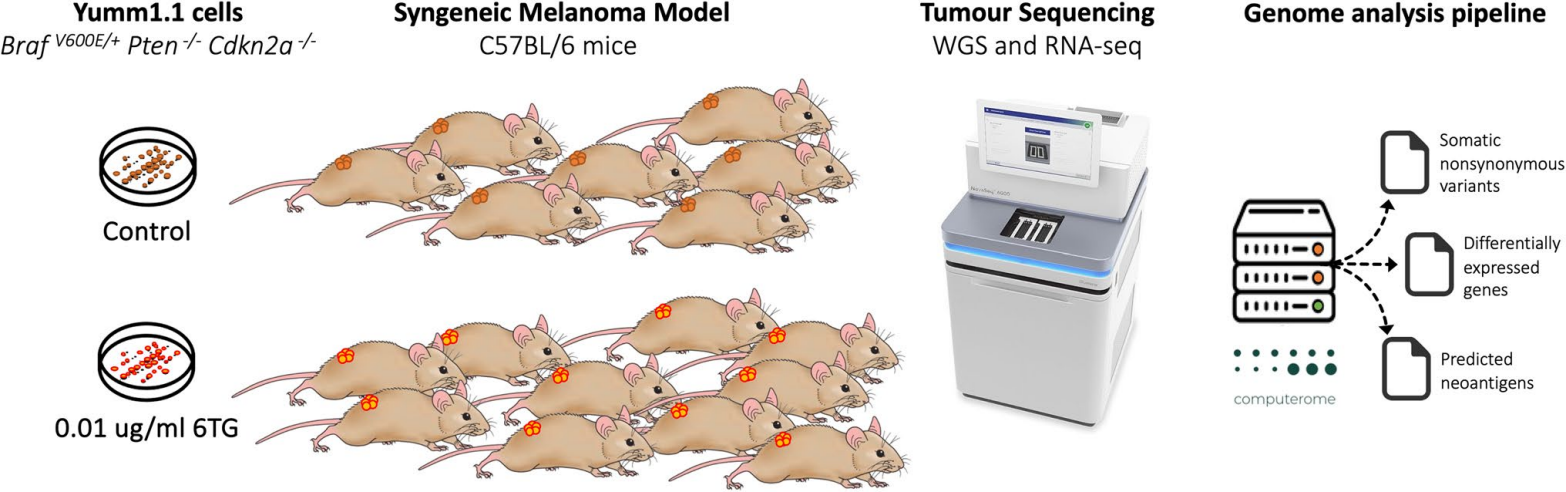
Schematic representation of the experimental design. Syngeneic melanoma tumours from control and treated samples were sequenced to study the 6TG effects by means of the mouse tumour genome analysis pipeline

In our previous study, 6TG was used to induce random mutations with the aim of increasing the number of neoantigens presented by tumour cells and improving the activation of the adaptive immune system [11]. Here, Yumm cells [24] were cultured in the presence or not of 6TG for a week. Hereafter, the pre-treated Yumm cells were injected subcutaneously into the right flank of immunocompetent C57BL/6N mice. We set out different in vivo experiments to monitor the progression of tumours [11]. To evaluate the mutational load in the murine tumours upon 6TG treatment, we performed WGS and RNA-seq of Control-Yumm derived tumours (control) and 6TG-Yumm derived tumours (6TG treated) bearing mice (Fig. 1). We used the GRCm38/mm10 mouse reference genome as C57BL/6 was the strain used to generate the mouse tumour model [11]. To compare and analyse the genetic profile and TMB of both groups, we developed the presented pipeline, and set out to monitor the in vivo progression of both groups of tumours.

**Fig. 2:**
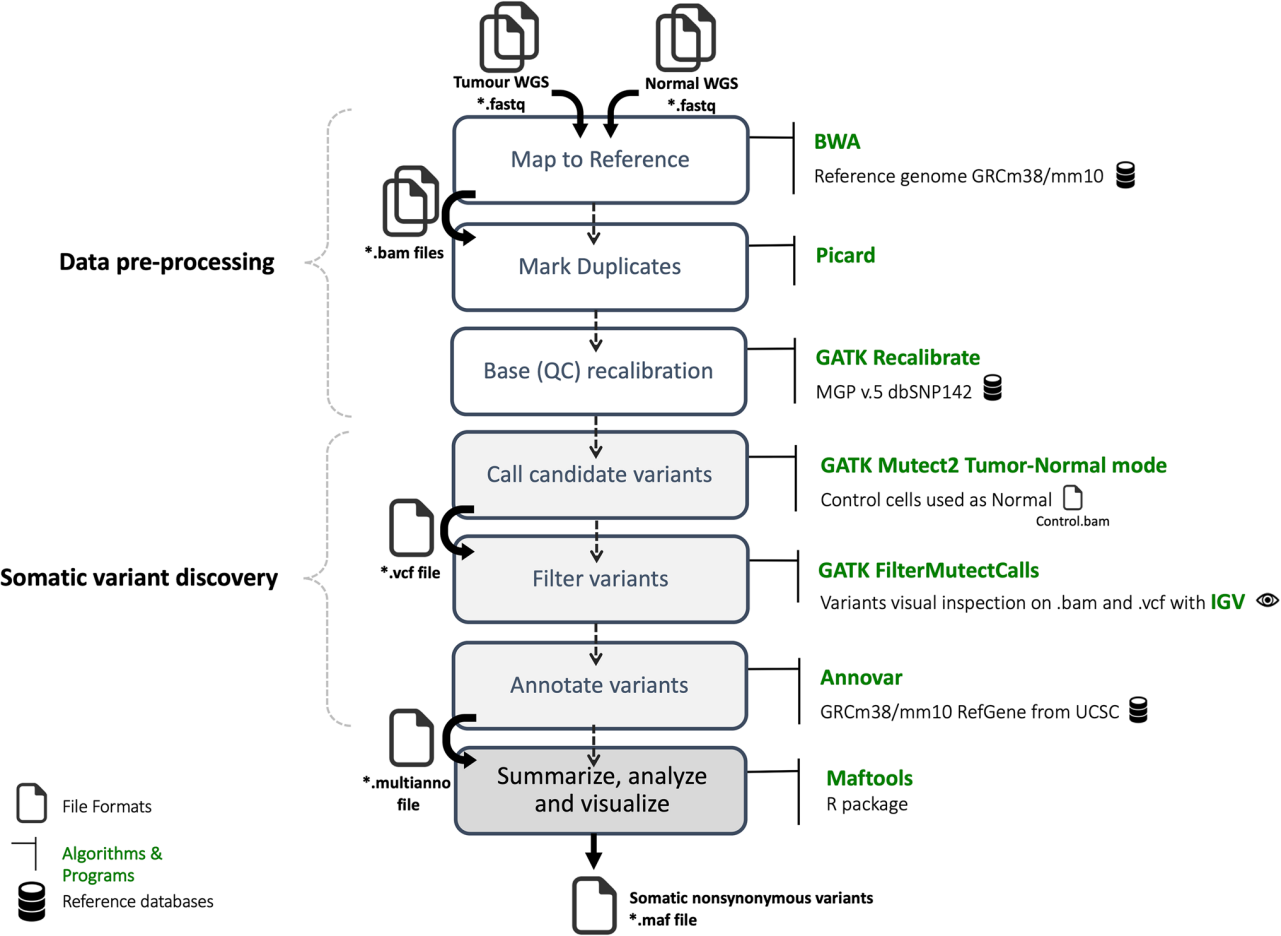
Mouse tumour WGS somatic variant discovery pipeline. Data pre-processing involves DNA reads alignment to reference (BWA), duplicates removal (Picard) and recalibration to know polymorphic sites (GATK). Somatic variant discovery involves somatic variant identification (GATK), filtering (GATK) and annotation (ANNOVAR). Final statistics and visualisation are performed with Maftools, visual inspection of findings is performed with IGV

**Fig. 3:**
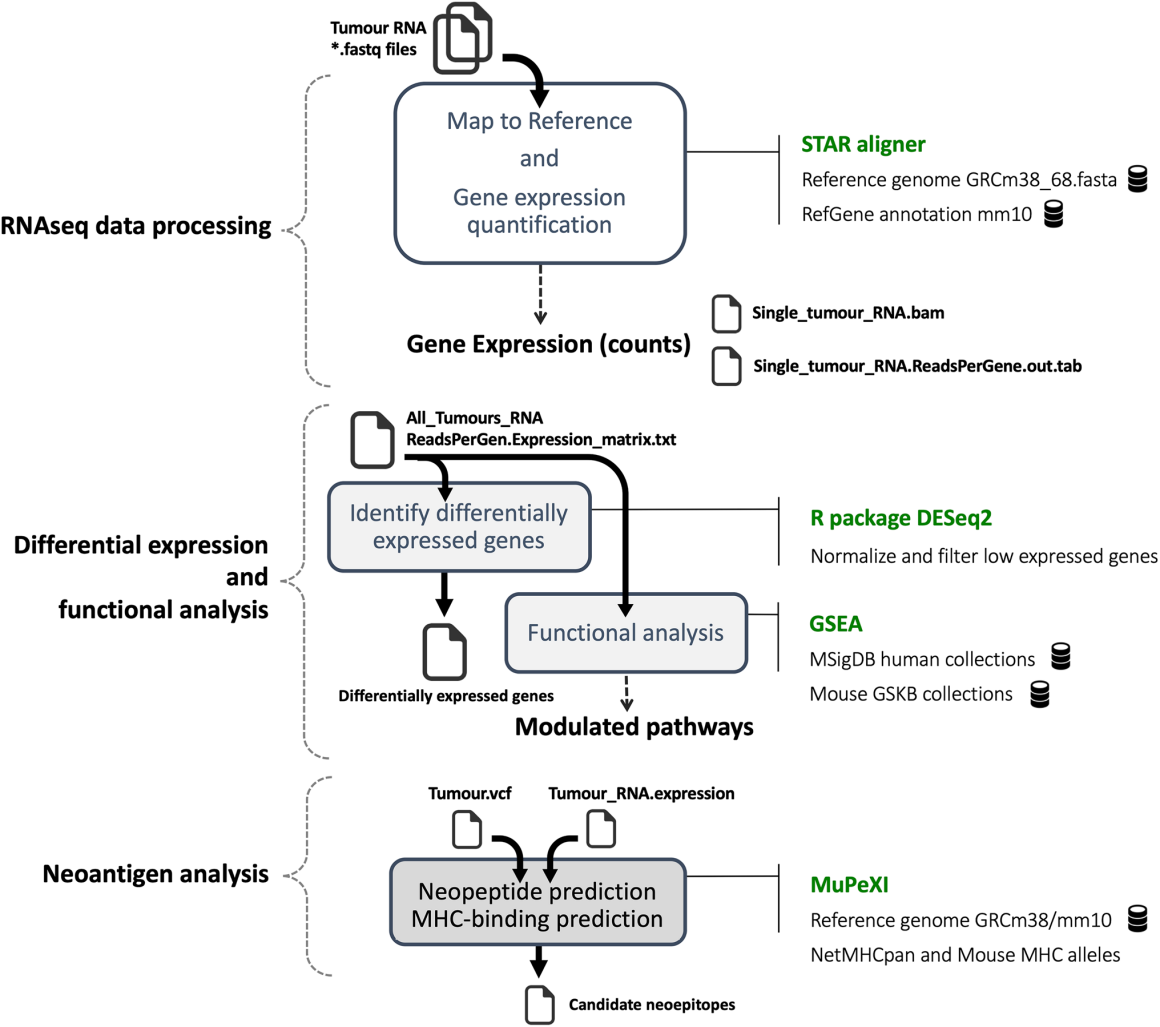
Mouse tumour RNA-seq data analysis and neoantigen prediction pipeline. RNA-seq data processing involves RNA reads alignment to reference and quantification (STAR). Differentially expressed genes were identified with DESeq2, and gene sets functional analysis were performed with GSEA. Finally, the neoantigen analysis includes neopeptide prediction and MHC binding analysis (MuPeXI)

Our pipeline was implemented on Computerome (https://www.computerome.dk/), the Danish National Computer for Life Science, and can be easily re-implemented on any Linux workstation. The pipeline includes i) WGS data analysis flow for mouse tumour somatic variant discovery (Fig. 2), ii) RNA-seq data analysis flow for gene expression analysis, and iii) neoantigen analysis flow for neopeptide prediction and MHC antigen presentation evaluation (Fig. 3). Although most of the methods included in our pipeline do not allow for parallel execution, the high-performance computing capabilities of Computerome let us run multiple workflows in parallel; hence making the analysis of multiple samples far more time efficient.

### WGS data analysis in 6TG-treated Yumm melanoma model

To study the TMB, which is defined as the number of somatic non-synonymous mutations per megabase (mut/Mb) of coding regions of a tumour genome, in the mouse tumours upon 6TG treatment, we extracted DNA from tumours at the endpoint time and performed WGS. We applied the mouse tumour WGS somatic variant discovery workflow to analyse raw fastq data files from 6TG-Yumm and Control-Yumm tumours samples. The WGS workflow takes ~ 7 days to conclude, being the alignment and the variant calling the more time-consuming tasks. After pre-processing the sample fastq files, somatic variants were identified with GATK Mutect2 set to tumour-normal mode using the non-treated control Yumm cells as normal. Then, we used the maftools R package to obtain statistics on variants and TMB. The resulting information is shown in Table 1.

**Table 1:** Statistics for identified variants, affected genes, tumour volumes, TMBs, potential neopeptides and predicted neoepitopes

Sample	Total variants	Median	Silent variants	Non-synonymous variants	Affected genes	Tumour volume (mm3 at day 28)	Median	TMB (mut/Mb)	TMB Median	Potential neopeptides	Median	Predicted neoepitopes	Median
Control-Yumm 1	20,165	14,049	19,891	274	270	98	556	5.48	3.34	10,416	6,171	817	439
Control-Yumm 2	16,143		15,976	167	163	655		3.34		6171		439	
Control-Yumm 3	10,252		10,139	113	112	556		2.26		4386		276	
Control-Yumm 4	10,367		10,240	127	125	730		2.54		5068		400	
Control-Yumm 5	14,049		13,880	169	166	513		3.38		6701		410	
Control-Yumm 6	35,668		35,233	435	423	907		8.7		16,737		1140	
Control-Yumm 7	13,732		13,593	139	135	505		2.78		5738		479	
6TG-Yumm 1	22,345	19,926	22,063	282	273	295	264	5.64	4.21	11,172	8,412	691	624
6TG-Yumm 2	15,446		15,243	203	197	169		4.06		8225		640	
6TG-Yumm 3	22,559		22,341	218	211	111		4.36		8600		607	
6TG-Yumm 4	10,825		10,694	131	131	386		2.62		5276		358	
6TG-Yumm 5	45,976		45,373	603	581	65		12.06		23,272		1487	
6TG-Yumm 6	12,484		12,343	141	134	539		3.54		5376		406	
6TG-Yumm 7	17,507		17,330	177	173	386		2.82		6871		523	
6TG-Yumm 8	39,637		39,150	487	470	135		9.74		19,176		1311	
6TG-Yumm 9	33,390		33,057	333	321	334		6.66		12,773		883	
6TG-Yumm 10	10,183		10,079	104	103	232		2.08		3890		246	

Control-Yumm are untreated samples, and 6TG-Yumm treated samples. Tumour volumes were collected at day 28 post injection. Neoepitope prediction results were obtained by filtering the potential neopeptides with best binding affinity score (MHCrank < 5) and existing gene expression (expression level > 0)

**Fig. 4:**
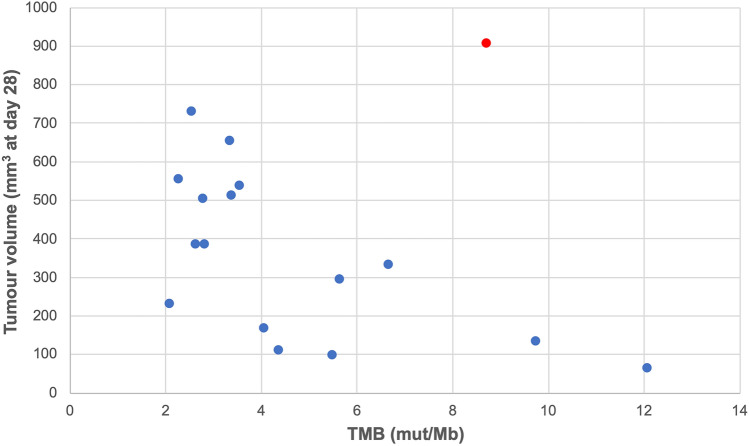
Tumour volume as a function of the TMB in each sample. Tumour volumes were collected at day 28 post injection. The identified outlier is highlighted in red

The WGS sequencing data analysis statistics revealed that the somatic variants median in Control-Yumm tumours was 14,049 compared to a median value of 19,926 found in 6TG-Yumm tumours, representing an increase of 41% in the number of variants. Although the number of variants was higher in 6TG-Yumm tumours, other statistics on mutation types and mutation allele frequencies showed no significant changes (data not shown). Accordingly, the median number of affected genes was increased by 25% in the 6TG-Yumm tumours. The monitoring of the in vivo progression of tumours and the TMB obtained showed that tumours with higher TMB had improved tumour control evaluated by the tumour volumes [11]. Investigating the relation between TMB and tumour volume, an overall negative correlation was apparent (Fig. 4). However, a clear outlier sample was identified (Control-Yumm 6) with highly abnormal tumour volume. Investigating the raw data for this sample revealed that this tumour volume resulted from the measurement of two tumours that developed adjacent to each other, thus suggesting that the volume was overestimated. Excluding this sample from the analysis, the correlation between TMB and the tumour volume was increased from − 0.30 to − 0.62 (*p* value < 0.005). The median TMB of Control-Yumm tumours was 3.34 mut/Mb; whereas, the median TMB of 6TG-Yumm tumours was 4.21 mut/Mb, representing a 26% increase. These results are consistent with earlier clinical research linking increased TMB to higher neoantigen levels and improved tumour control [25].

### RNA-seq data analysis in 6TG-treated Yumm melanoma model

**Fig. 5:**
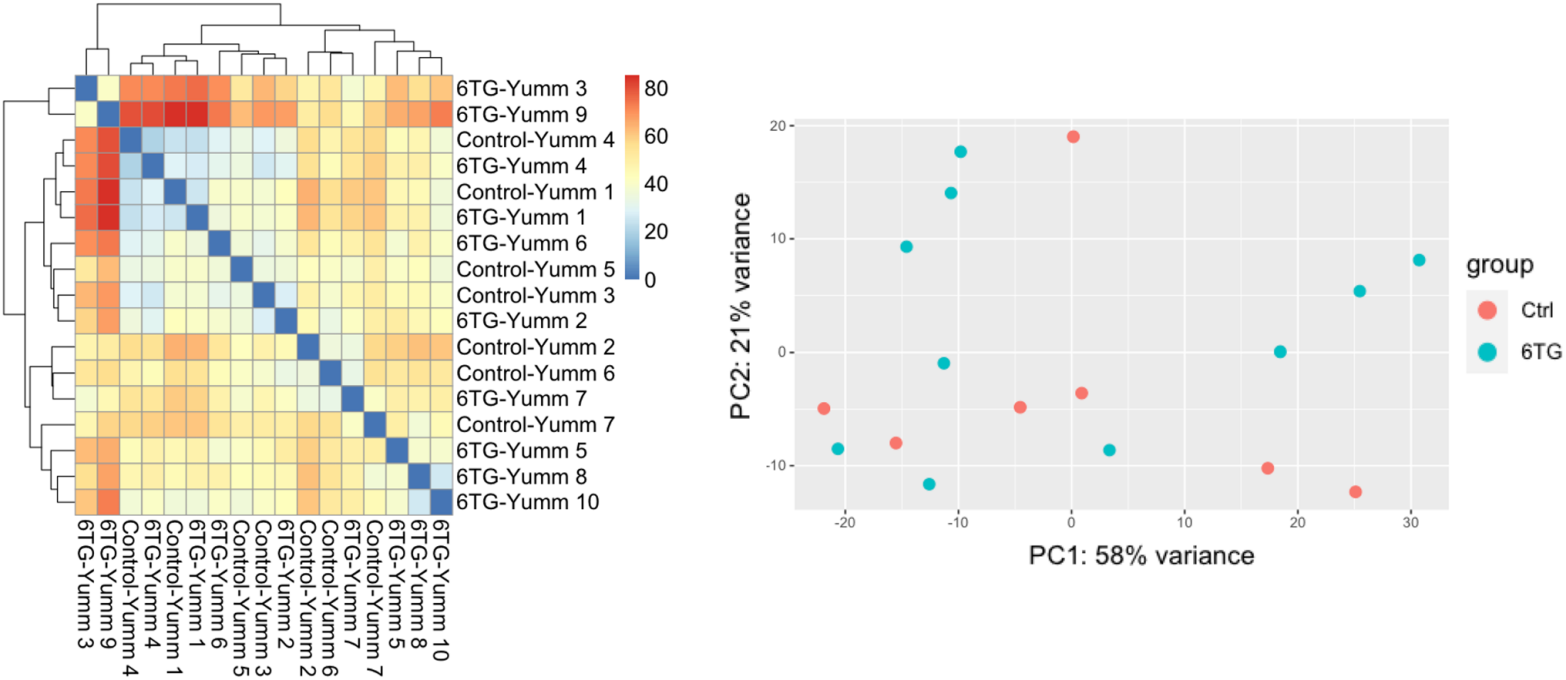
DESeq2 sample distance heatmap and PCA plot. On the left, the distance matrix and the automatic clustering results show gene expression profiles from different groups (Control and 6TG) are not clustered. On the right, the PCA plot show group gene expression profiles are not clustered

We processed Control-Yumm and 6TG-Yumm tumours RNA-seq data with the STAR aligner and then used the DESeq2 R package for gene expression analysis. Genes with no or very little expression (total read counts < 50) were filtered out, and read counts were normalised. The DESeq2 fitted statistical model found no genes differentially expressed (adjusted *p *value < 0.05) for 6TG-Yumm vs Control-Yumm condition. To further acknowledge the changes in gene expression, we calculated the sample distance matrix and PCA plots of all samples (Fig. 5). The clustering and the PCA plot showed similar results with Control-Yumm and 6TG-Yumm samples intermixed, as samples of different phenotypes display very similar expression profiles. These results suggest that the 6TG effect may cause no significant changes in gene expression of treated samples.

After processing all samples, we generated a gene expression matrix to perform functional analysis. Genes with low-counts were filtered prior to performing the gene set enrichment analysis (GSEA). The final expression dataset includes 15,998 genes. Enrichment analysis was performed on the mouse gene set collections from the Molecular Signatures Database (MSigDB v2022.1.Mm), and gene set database for pathway analysis in mouse from the Gene Set Knowledgebase (GSKB). These curated collections include positional, ontology, regulatory target genes, pathways and other gene sets. The GSEA analysis with permutation type parameter set on phenotype showed no statistically enriched gene sets (FDR < 25%) when comparing 6TG-Yumm expression to Control-Yumm expression, though a small number of gene sets were significantly enriched at nominal *p* value < 1% (data not shown). Although no statistically significant gene sets were found between the two phenotypes, as expected from the DESeq2 results, we ran a different second GSEA analysis with the parameter permutation type set to gene set, and not to phenotypes. In this way, by comparing among gene sets and not phenotypes, we were able to identify several important modulated pathways in 6TG-Yumm samples with FDR < 25% and nominal *p* value < 1%. The inflammatory response, immune receptor activity, myeloid leukocyte activation, cytokine activity and chemokine activity gene sets were among the most differentiated [11]. Cytokines and chemokines play critical roles in modulating the recruitment of T cells and the overall cellular composition of the tumour microenvironment [26].

### Neoantigens analysis in 6TG-treated Yumm melanoma model

The neoantigen analysis flow includes neopeptide discovery and MHC antigen presentation evaluation, which are important components in most vaccine and cancer research pipelines. The tumour neopeptide discovery and posterior neoepitope filtering were performed with MuPeXI. The number of tumour potential neopeptides and predicted neoepitopes are shown in Table 1 along with the tumour volumes collected at day 28 post injection.

**Fig. 6:**
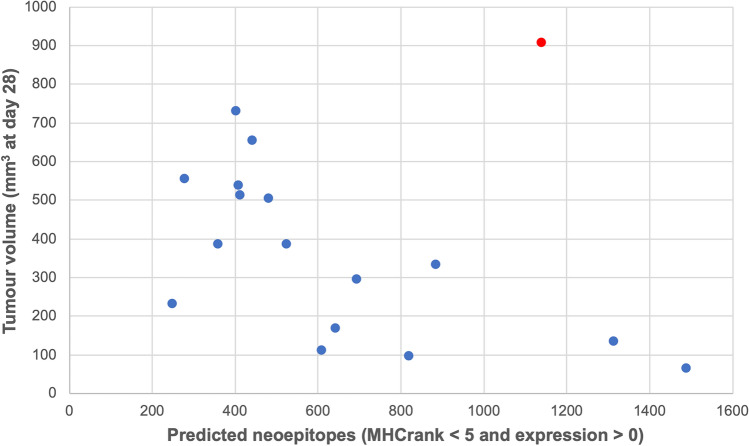
Tumour volume as a function of the number of predicted neoepitopes in each sample. Tumour volumes were collected at day 28 post injection. The identified outlier is highlighted in red

3.4. The in vivo experiments presented in Nazerai et al*.* showed an enhanced immune response as well as a lower volume of the 6TG treated tumours [11]. Excluding the outlier sample Control-Yumm 6, the median volume of the Control-Yumm tumours is 535 mm^3^ while the median volume of the 6TG-Yumm tumours is 264 mm^3^, representing a decrease in size of 51% (Table 1). The Control-Yumm tumours median of neoepitopes was predicted in 439 peptides while the median of 6TG-Yumm tumours was predicted in 624 peptides, representing an increase of 42% in the total number of predicted neoepitopes (Table 1). Further, the overall correlation between the tumour volume and number of predicted neoepitopes was found to be − 0.65 (*p* value < 0.005) (Fig. 6), a slight increase compared to the correlation found using the TMB. Considering this and that the median TMB of Control-Yumm tumours was 3.34 and the median TMB of 6TG-Yumm tumours was 4.21, which represent an increase of 26%, we can postulate that the amount of predicted neoepitopes is a more accurate estimator of tumour immune control compared to TMB.

All together our results indicate that 6TG indirectly increases the levels of neoantigens presented by tumour cells which results in an improved tumour control. Our strategy of performing in vitro and in vivo experiments combined with the development of a genome analysis pipeline for mouse tumours allowed for carrying out a detailed characterisation of the 6TG effects on tumour cells, and let us implement a robust genome analysis platform for future mouse genomic analysis.

## Discussion

In this study, we present a genome analysis pipeline for mouse tumours developed following the best practices. The WGS data analysis flow for somatic variant discovery, the RNA-seq data flow for differential expression analysis, and the neoantigen analysis are all based on standards, and can be easily replicated in other Linux workstations. The turnaround times of WGS data analysis pipelines are usually long and runtimes are measured in days of processing. This is due to the massive amounts of data that needs to be processed added to the fact that most analysis algorithms cannot be used in parallel or with distributed computing yet.

With the use of the presented state-of-the-art mouse genome analysis pipeline and the in vitro and in vivo experiments we were able to demonstrate that treatment of Yumm cells with 6TG can markedly enhance the immune response in our pre-clinical melanoma model and promote the efficacy of ICI therapy [11]. The WGS/RNA-seq data analysis and in vivo experimental results showed that the applied dose the 6TG is sufficient to increase the TMB, the levels of neoepitopes, and improve the immune response. The RNA-seq data analysis found no statistically significant differentially expressed genes between 6TG treated and control phenotypes, but the functional analysis comparing the different gene sets identified some important immunogenic pathways modulated by 6TG treatment. Our findings are promising and provide proof of concept for the clinical use of low doses of 6TG in addition to the use of mercaptopurine and ICI therapies for malignancies with low TMB that are unresponsive to conventional therapies. In light of our results, we are currently conducting a phase I/II clinical research to investigate the potential of thiopurine treatment in increasing the proportion of otherwise treatment-resistant cancer patients who may derive therapeutic benefits from ICI therapy (clinicaltrials.gov: NCT05276284).

### Supplementary Information

Below is the link to the electronic supplementary material.

Supplementary file1 (SH 8 KB)

## Data Availability

The WGS and RNA-seq datasets generated and analysed during the current study will be available from the corresponding authors upon request. The datasets are currently deposited on Computerome, the Danish National Computer for Life Sciences, https://computerome.dtu.dk
